# Understanding the Epigenetic Landscape of Barrett's Esophagus and Esophageal Cancer: A Review of Current Findings, Caveats and Future Directions

**DOI:** 10.1002/gcc.70167

**Published:** 2026-08-31

**Authors:** Louise Lawless, Trevor Doherty, Carla Surlis, Niamh Gilmartin, Therese M. Murphy

**Affiliations:** ^1^ Sustainability and Health Research Hub, Greenway Hub, Technological University Dublin Dublin Ireland; ^2^ School of Biological, Health and Sports Sciences, Technological University Dublin Dublin Ireland; ^3^ Research Ireland Centre for Research Training in Machine Learning, Technological University Dublin Dublin Ireland

**Keywords:** Barrett's esophagus, DNA methylation, epigenetics, esophageal cancer

## Abstract

Esophageal cancer (OC) is currently the eighth most common form of cancer worldwide with a 5‐year survival rate of 10%–20%, with the primary risk factor of esophageal adenocarcinoma (OAC) being the development of Barrett's Esophagus (BO). Despite its clinical significance, the molecular pathogenesis underlying both BO and OC is not well understood. In recent years, epigenetic dysregulation, particularly aberrant DNA methylation, has emerged as a critical area of investigation, given its potential utility in the identification of diagnostic, prognostic, and therapeutic biomarkers. This review examines the evolving epigenetic landscape of esophageal cancer, including a focus on its origins in BO with a particular emphasis on DNA methylation, the most extensively researched epigenetic mechanism. Key DNA methylation‐associated alterations involved in OAC and OSCC initiation and progression are discussed, alongside their potential clinical application as biomarkers for early detection, prognosis, and risk stratification in BO populations. Furthermore, the role of these epigenetically regulated genes in the disruption of Wnt signaling and cell cycle control pathways implicated in esophageal carcinogenesis is explored. The review concludes by outlining future research directions, current challenges, and the promise of epigenetic studies in advancing our understanding of OC pathogenesis and improving patient outcomes.

## 1 Introduction

Esophageal cancer (OC) is currently the eighth most common form of cancer worldwide [1].

The incidence rate is rapidly increasing, with a sixfold increase reported in recent years [2]. OC primarily includes two main subtypes: squamous cell carcinoma (OSCC) and adenocarcinoma (OAC), each with distinct molecular characteristics, risk factors, and clinical outcomes [3]. While OSCC is most common, OAC rates are rising in Western countries, predominantly in Europe, the Americas, and Australia [1]. The causes for the rise in cases are generally unknown; however, it has been speculated that the upsurge could be due to increasing prevalence of risk factors such as obesity and heartburn [4]. All subtypes of OC have a high mortality rate and despite improvement in treatments, the 5‐year survival rate remains 10%–20% [2, 5]. High mortality rates are mostly linked to the absence of early clinical symptoms and OC typically being detected in later stages when treatment options are reduced [3]. The primary risk factor for developing OAC is the presence of Barrett's Esophagus (BO) [6], a complication of gastroesophageal reflux disease (GERD) [3]. BO is a premalignant condition occurring when the normal squamous epithelium in the esophagus is transitioned to columnar lined cells [6], like those found in the intestines. Approximately 6%–14% of patients with GERD develop BO, of whom approximately 0.5%–1% will subsequently develop OAC [7].

Endoscopy and biopsy are considered the gold standard for the initial diagnostics for OC [8], but due to the invasive nature of OC and variation associated with histological diagnosis, genetic and epigenetic‐based diagnostic biomarkers may serve to improve risk stratification, diagnosis, and prognosis [9]. This review will provide a concise overview of epigenetic mechanisms, with a particular emphasis on DNA methylation, the most extensively researched epigenetic modification in OC. It will outline promising novel DNA methylation‐based biomarkers for BO and OC, and conclude with a discussion of future research directions, potential methodological challenges, and the promise of this field in advancing our understanding of OC.

## 2 Methods

**FIGURE 1:**
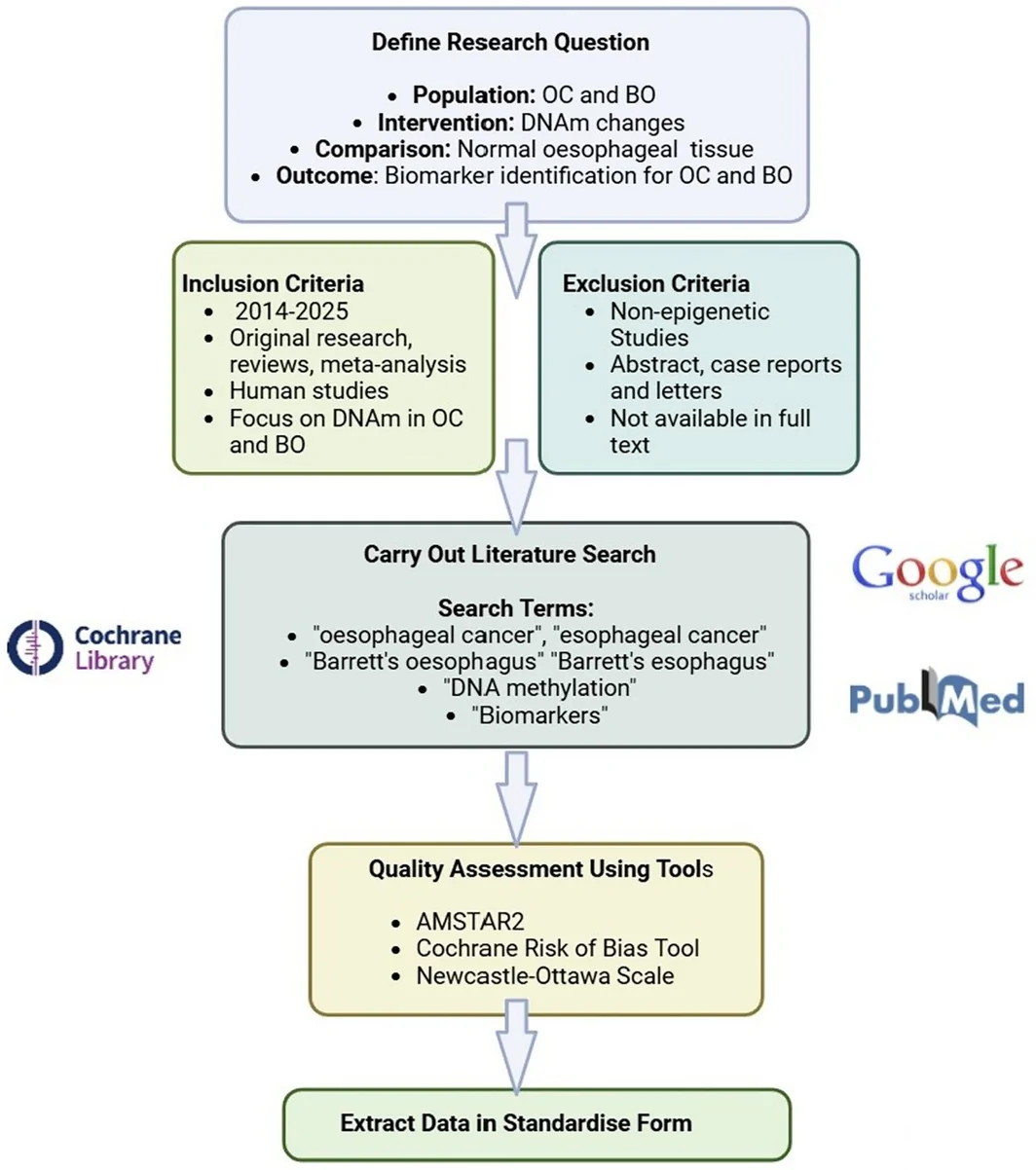
Workflow outlining methods for literature review. Created in BioRender (2025).

**FIGURE 2:**
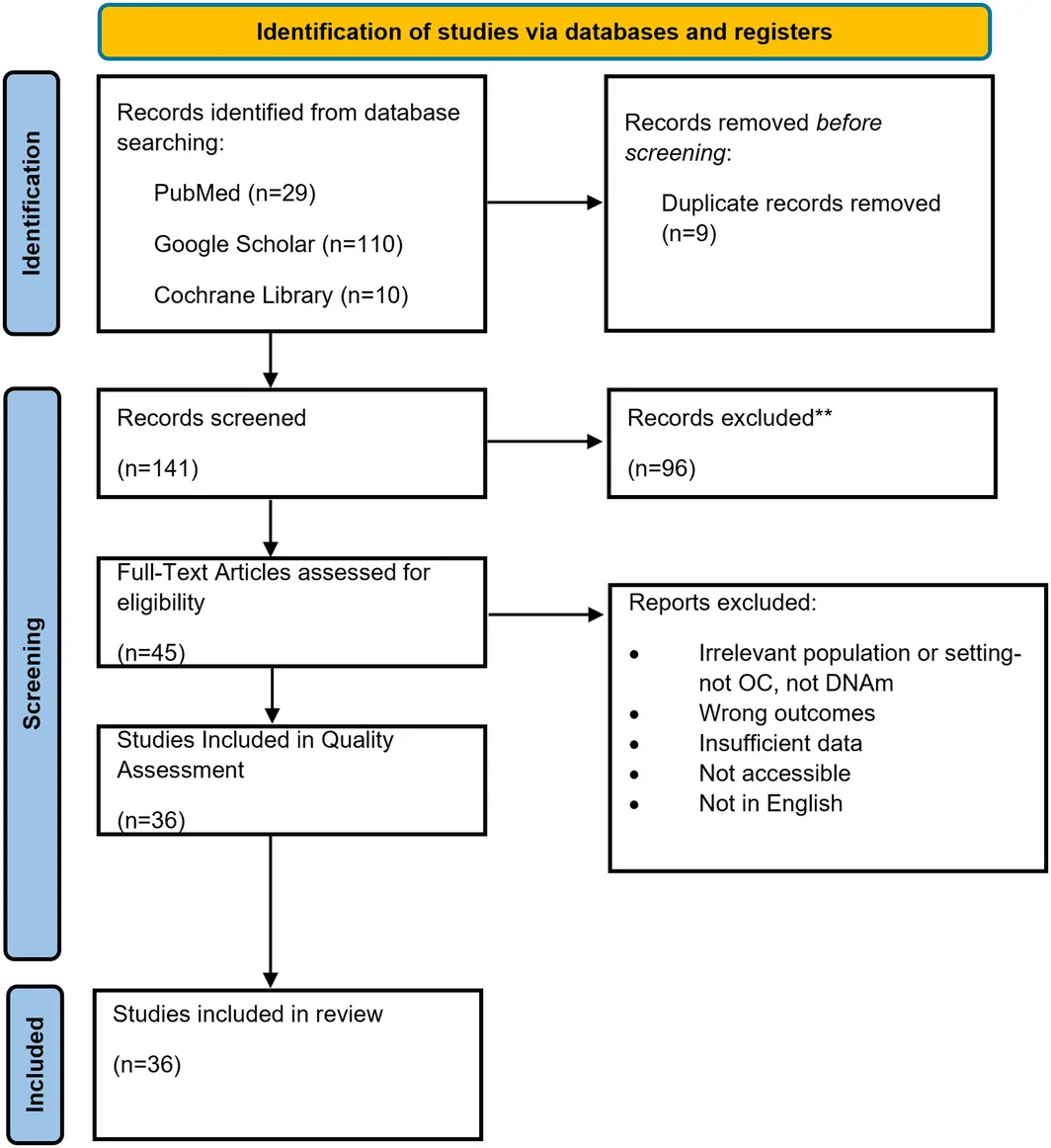
PRISMA flow diagram. Included, excluded and screened papers to identify frequently differentially methylated genes in OC and BO in comparison to non‐cancerous esophageal tissue.

Using the PICO framework, a research question was developed to identify key differentially methylated genes associated with OC in the literature. The databases PubMed, Google Scholar, and Cochrane Library were screened for articles relevant to key words such as “esophageal cancer”, “Barrett's esophagus,” and “DNA methylation”. Duplicate papers from the initial search were removed, and the remaining papers were assessed for relevance and quality and removed as appropriate. The remaining papers (*N* = 36) were screened, and the data was extracted in a standardized form. This data was used to determine genes differently methylated in OC and progression from BO to OAC compared to non‐cancerous esophageal tissue in published literature and has been summarized in Table 1. PRISMA was developed as a method to aid completion and accuracy in systematic reviews [10], and while screening data, the number of papers included (*N* = 36) and excluded (*N* = 96) were noted in a PRISMA flow diagram to ensure transparency and completion. A workflow of the methodology used to conduct the literature review is outlined in Figure 1. The completed PRISMA Flow Diagram of articles that were included, excluded, and screened for data is illustrated in Figure 2.

**TABLE 1:** 10 key genes frequently epigenetically dysregulated in esophageal cancer identified in the literature.

Gene	Hypo/hypermethylation	Chromosome region	Gene function (gene card)	Reported diagnostic potential	Methylation status in Barrett's Esophagus	Technologies used to determine methylation status	Ref (PMID)
*ZNF569*	Hypermethylation	Promoter: 19q13.12	Transcriptional regulation using RNA Polymerase II	Sensitivity: 53%–69.3% Specificity: 87%–99.2%	Hypermethylation	Cytosponge collection followed by Methylation Specific qPCR (MS qPCR), plasma derived circulating DNA followed by MS qPCR	33292587 36670548
*APC*	Hypermethylation	Promoter 1A: 5q21 region	Wnt signaling pathway, tumor suppressor	Sensitivity: 55% Specificity: 96%	Hypermethylation	MS qPCR, methylation‐sensitive high‐resolution melting, methylation‐sensitive dot‐blot assays and methylation ligation‐dependent arrays.	29440928
*CDKN2A*	Hypermethylation	Promoter: 9p21	Tumor suppressor gene	Sensitivity: 52%–81.8% Specificity: 96%–100%	Hypermethylation	Microdissection of FFPE tissue followed by methylation‐sensitive single‐strand conformation analysis (MS‐SSCA), MS qPCR	28637022 11910361
*TWIST1*	Hypomethylation	Promoter: 7p21.2	Transcriptional regulation and gene expression regulation	Sensitivity: N/A Specificity: N/A	Hypermethylation	MeDIP‐seq followed by bisulfite sequencing, MS qPCR	26683359
*SFRP1*	Hypermethylation	Promoter	Wnt signaling, cell growth and differentiation	Sensitivity: 92.5%–95% Specificity: 35%–90%	Hypermethylation	FFPE tissue followed by MS qPCR, fresh‐frozen tissue followed by MS qPCR	15825175 21567192
*MGMT*	Hypermethylation	Promoter: 10q26	Cellular defense against the biological effects of O6‐methylguanine (O6‐MeG) and O4‐methylthymine (O4‐MeT) in DNA.	Sensitivity: N/A Specificity: N/A	Hypermethylation	MS qPCR	18199718 16899597
*TP53*	Hypermethylation	17p13.1	Tumor suppressor gene	Sensitivity: N/A Specificity: N/A	Hypermethylation	SYBR Green MS qPCR	35725923
*PAX1*, *SOX1*, and *ZNF582*	Hypermethylation	*PAX1*: *20q13.12* *SOX1*: promoter: 13q12.3 *ZNF582*: promoter: 19q13.43	*PAX1* and *SOX1*: Transcription activator *ZNF582*: Transcription regulation	Sensitivity: 94.6% Specificity: 77%	*PAX1* and *ZNF582*: Hypermethylation	Tissue samples followed by PCR Pyrosequencing	31629253
*OTOP2* and *KCNA3*	Hypermethylated	*OTOP2*: promoter: 3p14.3 *KCNA3*: promoter: 1p34.3	*OTOP2*: proton selective ion channel *KCNA3*: Mediates potassium ion permeability in membranes	Sensitivity: 87.4% Specificity: 93.3%	No hypermethylation	Plasma derived cfDNA followed by multiplex MS qPCR	38890756
*SOX17*	Hypermethylation	Promoter: 8q11.23	Acts as a transcription regulator that binds target promoter DNA and bends the DNA and inhibits Wnt signaling	Sensitivity: N/A Specificity: N/A	Hypermethylation	Pyrosequencing methylation	30777052

*Note:* Sensitivity and specificity are reported where diagnostic performance was evaluated. Where available, values represent pooled estimates from meta‐analyses; otherwise, they are taken from individual biomarker or biomarker‐panel studies. Not all studies evaluated or reported sensitivity and specificity, and these entries are shown as N/A. Ranges represent results from separate studies. For combined biomarker panels, the reported performance should not be attributed to each constituent gene individually. Differences in study population, histological subtype, specimen type and methylation‐detection methodology should be considered when comparing results.

## 3 Epigenetic Modifications and Their Role in Cancer

Epigenetics describes the mechanisms that control gene expression and is defined as the study of mitotically (and meiotically) heritable alterations in gene expression that are not caused by changes in the DNA sequence [11]. Epigenetic modifications involve chemical alterations to the DNA sequence or histone proteins that surround the DNA, resulting in structural changes of the chromatin. Such epigenetic modifications can act like molecular switches, fine‐tuning how genes are expressed [12]. A combination of DNA methylation, histone modifications, and non‐coding RNAs work together to regulate gene expression in normal human cells [13]. Aberrant epigenetic patterns play a pivotal role in cancer development and progression by disrupting the normal regulation of gene expression, leading to the activation of oncogenes, silencing of tumor suppressor genes (TSG), and altering key cellular pathways involved in proliferation, survival, and metastasis [14], and identifying which genes are abnormally altered in OC may open pathways to new diagnostics and therapies.

### 3.1 DNA Modifications

DNA methylation (DNAm), the most thoroughly studied epigenetic modification in cancer, involves the addition of a methyl group to the 5‐carbon of cytosine residues, typically in the context of CpG dinucleotides [15]. It plays a crucial role in regulating gene expression, maintaining genomic stability, and influencing cell differentiation and development [16]. Carcinogenesis is associated with global DNA hypomethylations, promoter hypermethylation and frequent deamination events, which contribute to tumourigenesis through various mechanisms such as the activation of oncogenes (through DNA hypomethylation), silencing of TSGs (through hypermethylation) and silencing of DNA repair genes [16]. DNAm is catalyzed by DNA methyltransferases (DNMTs), DNMT1, 2, 3A, 3B, and 3L, which each play a separate role in the DNA methylation process. Overall, DNAm is a critical epigenetic process in cancer progression, initiation, and maintenance [15].

### 3.2 Other DNA Modifications

Besides DNAm, other epigenetic modifications include hydroxymethylation which is linked to active gene regulation and carcinogenesis. 5‐Hydroxymethycytosine (5hmC) is an epigenetic modification that is derived from the 5‐methylcytosine (5mC) which is generated by the ten eleven translocation (TET) enzymes that oxidize 5mC to 5hmC [17]. This modification is thought to play a role in gene activation [17]. In cancer, levels of 5hmC are lower compared to normal tissue and, alongside the dysregulation or loss of function of the TET enzymes, this indicates that the dysregulation of 5hmC plays a role in tumourigenesis [18], and therefore could potentially be used as a general cancer biomarker [19].

### 3.3 Histone Modifications and Other Epigenetic Mechanisms

Histone modifications are post‐translational chemical alterations of histone proteins, including histone methylation and acetylation. They play a crucial role in the regulation of the chromatin structure and gene expression and therefore impact processes such as DNA repair, transcription, and replication [20]. These regulators allow for accurate and dynamic control of gene expression in response to disease states and environmental and developmental stimuli and in cancer, abnormal histone modifications can lead to the silencing of tumor suppressor genes or activation of oncogenes, contributing to uncontrolled cell growth and tumor progression [20].

Non‐coding RNAs (ncRNAs), such as microRNAs (miRNAs) and long non‐coding RNAs (lncRNAs), are RNA molecules that are transcribed from DNA but do not code for proteins [21]. ncRNAs play a role in regulatory processes including gene expression, epigenetic regulation, and chromatin remodeling [21]. In cancer, dysregulation of ncRNAs can disrupt normal cellular processes, promoting tumor growth, metastasis, and resistance to therapy [22].

In recent years, epigenetics has been a major topic of interest in oncology research and has shown enormous potential in the areas of therapeutics, diagnostics, and prognostics [23]. The most common form of epigenetic alteration in cancer is aberrant DNA methylation, and this has shown potential for diagnostic and prognostic markers in solid tumors [24]. Changes to genes, such as TSGs and oncogenes, can occur from one or a combination of epigenetic modifications, loss of heterozygosity (losing a part of or a copy of a gene) or alteration to the coding sequence [13, 23]. These changes can result in pathways and processes occurring abnormally, such as cell division, angiogenesis, and apoptosis [25]. With epigenetic changes possibly being reversible and malleable, this makes them an important target for therapeutics in oncology [13].

## 4 Epigenetic Dysregulation in BO and OC

While the molecular mechanisms associated with OC are still being studied, it is widely accepted that epigenetic changes have a major influence on the development of OC [26].

Therefore, deciphering the epigenetic landscape of OC, and its precursor BO, has the potential to aid in the discovery of diagnostics, prognostics, and markers of disease progression and response to therapy in OC [27]. It may also aid in the development of targeted epigenetic‐based therapeutics for OC. Over the past decade, several promising DNA methylation‐based biomarkers of OC have been identified. Because early detection remains a major challenge in OC, consolidating evidence on epigenetically altered genes is critical for advancing biomarker development. In Table 1, we highlight key genes (and their function) that are frequently epigenetically dysregulated in OC.

### 4.1 DNA Modifications in OC and BO

#### 4.1.1 DNAm Biomarkers for Differentiating OC From Normal Esophageal Tissue

Tang and colleagues identified a potential DNA methylation panel with *PAX1*, *SOX1*, and *ZNF582*, with this panel detecting OSCC with a sensitivity of 94.6% and a specificity of 77% compared to normal tissue [28]. There were slight variations in DNAm status across patient samples and genes. *ZNF582*, for example, had 100% in both sensitivity and specificity in females, while it attained a 94% sensitivity and 74.6% specificity in males [28]. *ZNF582* also had lower methylation levels in well‐differentiated OC tumors [28]. *SOX1* DNAm levels also differed depending on the location of the tumors, with upper esophageal tumors showing 48.5% *SOX1* DNAm and lower esophageal tumors having a mean DNAm of 18.16%. While the exact reason for DNAm variation between locations is unknown, it is thought to be associated with the level of mucosal damage [28].

These studies show promise that not only can OC be diagnosed through an appropriate DNA methylation panel, but we will be able to further subtype OSCC which will aid in clinical outcomes [27].

#### 4.1.2 DNAm Biomarkers for Differentiating OAC From OSCC

Comprehensive molecular profiling has demonstrated that OAC and OSCC are distinct disease types with markedly different molecular characteristics, including differences in their epigenetic landscapes [29]. Nevertheless, some DNA methylation alterations, including hypermethylation of tumor suppressor genes such as *CDKN2A*, have been reported in both subtypes, suggesting that while subtype‐specific biomarkers are required, certain epigenetic changes may reflect common mechanisms of esophageal carcinogenesis [30]. Specifically, *CDKN2A* is found to be hypermethylated in both subtypes [30]. As *TP53* and *CDKN2A* regulate distinct but complementary cell‐cycle pathways, alterations affecting these genes may collectively contribute to esophageal carcinogenesis [30]. To overcome the issue of differing subtypes, Pu et al. developed a five‐marker panel to specifically identify OSCC compared to OAC tissue including three promoter regions, *STK3*, *ZNF418*, and *ZNF542* and two CpG sites cg19396867 and cg20655070 [31]. In a further study by Lu et al., a five gene DNA methylation panel (*ABCD1*, *SLC5A10*, *SPIN3*, *ZNF69*, and *ZNF608*) was identified to distinguish between OAC and OSCC [32].

#### 4.1.3 DNAm Biomarkers and Processes in the Transition From Barrett's Esophagus to Esophageal Adenocarcinoma

Identifying DNA methylation‐based biomarkers for OC and BO is currently an active area of research. Studies have produced gene panels such as those from Alvi and colleagues, who produced a four gene panel consisting of *PIGR*, *RIN2*, *GJA12*, and *SLC22A18* [33], which was subsequently validated in a prospective cohort to stratify patients and was found to have specificity of 97% and a sensitivity of 94% for distinguishing between OAC/high grade dysplasia and BO [33]. Another study by Jin and colleagues reported a panel incorporating *p16*, *HPP1*, *RUNX3*, *NELL1*, TAC1, *AKAP12*, and *CDH13* which demonstrated potential for predicting progression from BO to OAC [34]. These and other models were comprehensively reviewed by Nieto and colleagues, who concluded that despite encouraging results, prospective validation and methodological standardization remained necessary before clinical implementation [35].

It is important to understand the processes and epigenetic links with the transition from BO to OAC to develop early biomarker panels for diagnostic and prognostic applications [36]. The progression of BO to OAC is a process that can take many years and can be caused by a multitude of factors [37]. It is theorized that the epithelium of the esophagus, through GERD damage, can gain characteristics of progenitor cells and this allows them to change their phenotype from normal squamous epithelium to the columnar epithelium that is seen in BO [38]. Chronic inflammation due to GERD contributes to the epigenetic alterations and fosters a microenvironment supportive of cancer progression [38]. Many tumor suppressor genes that are commonly found in cancer are hypermethylated in OC and BO, such as *MGMT* and *CDKN2A* (see Table 1 for further details) [39]. *TP53* promoter methylation has also been reported in OC [40], however, its potential as a marker of dysplastic progression is more strongly associated with genetic alterations, particularly *TP53* mutation and loss of heterozygosity (LOH), which occur during neoplastic progression from BO to esophageal adenocarcinoma [41]. In BO, there is often an increase in DNA methylation at specific CpG islands located in the promoter regions of TSGs such as *CDKN2A*. Research suggests *CDKN2A* is one of the first genes aberrantly methylated in the transition from BO to OAC [9], making it a gene of interest for early OC detection, as highlighted in Table 1. Many of the epigenetic and genetic alterations identified in BO are also found in OC, supporting their potential clinical use in assays assessing the risk of progression from BO to OAC [41]. For example, *TWIST1* and *ZNF569* were found to be differently methylated in both BO and OC, with a study by Chettouh et al. identifying *TWIST1*, *ZNF569*, *ZNF345*, and *TFPI2* as a potential DNA methylation panel for the detection of BO [42]. Caution is required when examining differently methylated genes in BO as an indicator of disease progression as not all patients with specific DNAm patterns will go on to develop OC [43]. Table 1 highlights a panel of key genes that are differentially methylated in OC, showing their potential as epigenetic biomarkers for early detection. Notably, many of these genes show methylation changes not only in OC but also in BO, indicating that aberrant DNAm patterns emerge early in the neoplastic process. This overlap strengthens the case for their use in risk stratification and early diagnosis, providing an epigenetic link between precancerous changes and carcinogenesis.

### 4.2 Common Pathways Epigenetically Dysregulated in OC and BO

Examining the function of key genes frequently epigenetically dysregulated in OC and BO (see Table 1) has identified key molecular pathways which are frequently disrupted in OC. In particular, the Wnt pathway and cell cycle regulation emerged as consistently disrupted, highlighting their central roles in the epigenetic landscape of OC progression. These pathways can also be interpreted within the framework of the Hallmarks of Cancer proposed by Hanahan and Weinberg [44, 45], demonstrating how aberrant DNA methylation contributes to several fundamental biological capabilities acquired during tumor development, including sustaining proliferative signaling, evading growth suppressors, resisting cell death, enabling replicative immortality, promoting genomic instability, and facilitating invasion and metastasis [45]. Consequently, the epigenetic dysregulation of genes involved in these pathways not only provides mechanistic insight into the progression of BO to OAC but also highlights their potential clinical utility as diagnostic, prognostic and therapeutic biomarkers [44, 45].

#### 4.2.1 Wnt Signaling

**FIGURE 3:**
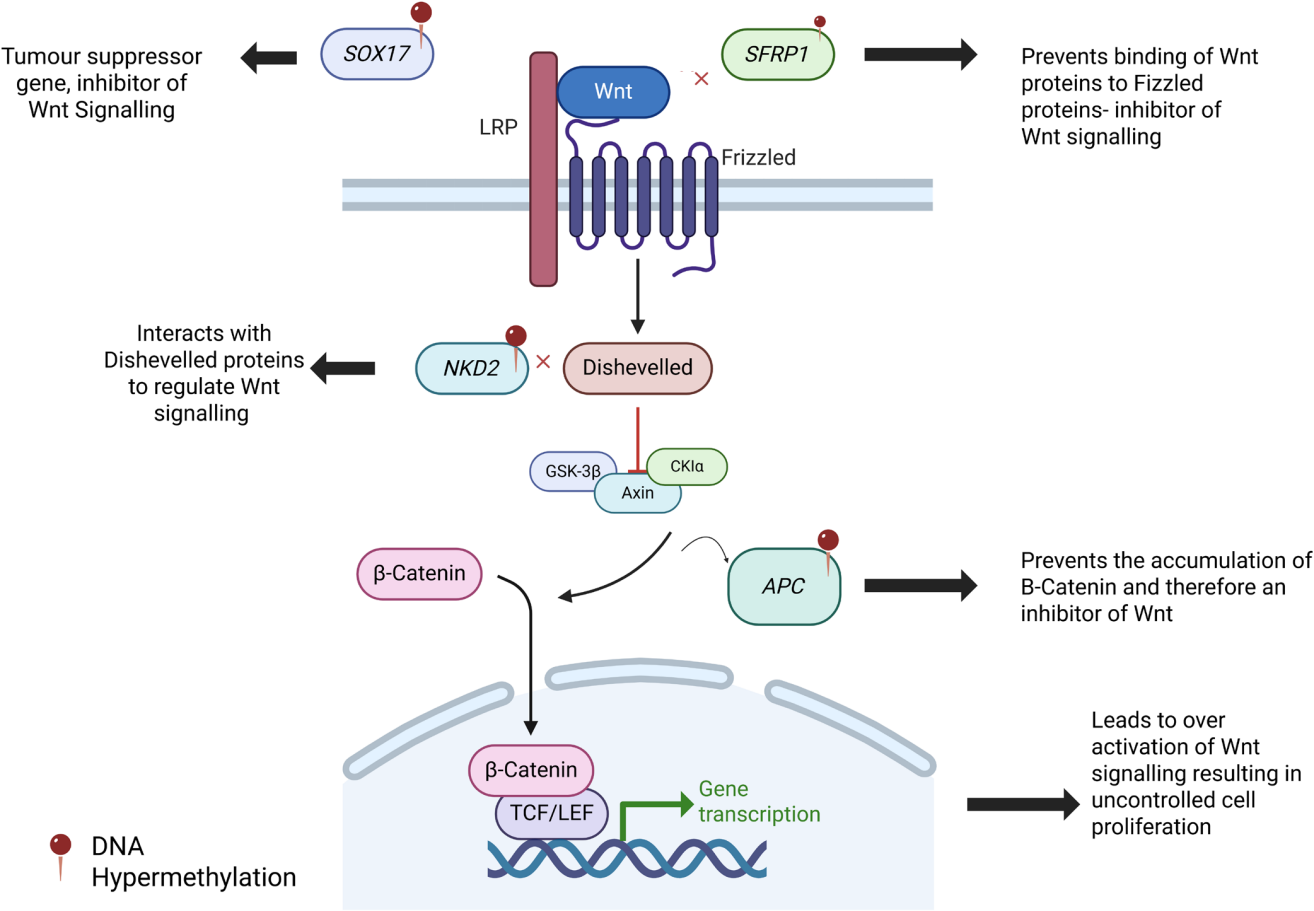
Wnt signaling cascade and the molecular pathways seen to be dysregulated in OC including SOX17, APC, NKD2 and SFRP1. Red lollipop symbols highlight genes that are aberrantly methylated in OC. Created in BioRender (2025).

The Wnt signaling cascade (Figure 3) is essential for homeostasis, regeneration and tissue morphogenesis and the dysregulation of this pathway promotes carcinogenesis through the malignant transformation of progenitor cells, causing increased proliferation [46] As shown in Figure 3, this pathway starts with Wnt proteins that are secreted glycoproteins that initiate the Wnt signaling cascade [47]. Upon Wnt binding to its receptor, Disheveled (a cytoplasmic protein) is activated [47]. This inhibits the destruction complex that normally degrades β‐catenin. In the nucleus, β‐catenin interacts with TCF/LEF (T‐cell factor/lymphoid enhancer factor) transcription factors, leading to the activation of Wnt target genes [47].

Several differentially methylated genes that have been implicated in the disruption of the Wnt signaling pathway have been linked to both OC and BO, including *APC* (adenomatous polyposis coli), *NKD2* (naked cuticle homolog 2), and *SFRP1* (secreted frizzled‐related protein [46]). The *APC* gene plays a crucial role in the Wnt signaling pathway, and, functioning normally, *APC* helps to regulate β‐catenin, preventing its accumulation and thereby inhibiting uncontrolled cell proliferation [48], as illustrated in Figure 3. *APC* is frequently hypermethylated in OC, as seen in Table 1, which contributes to the loss of its tumor suppressive functions, and DNAm changes in *APC* were found in BO samples and in approximately 95% of OAC cases [49].

*SFRP1* binds directly to the Wnt proteins and prevents binding to the Frizzled family receptors, therefore functioning as a secreted inhibitor of Wnt signaling [50]. *SFRP1* is in a chromosomal region that is frequently silenced in cancers, indicating that *SFRP1* plays a tumor suppressing role in carcinogenesis [50]. DNAm of *SFRP1* was found in BO samples and in an estimated 65% of OAC cases [51].

*NKD2* is a negative regulator of Wnt signaling and aids in suppressing tumor growth through the interactions with Disheveled proteins in the Wnt pathway [52]. Expression of *NKD2* is completely lost or greatly reduced in several OSCC lines and it is believed that *NKD2* contributes to tumourigenesis in OC by suppressing Wnt signaling in vitro and in vivo [46].

The tumor suppressor *SOX17* (identified as a relevant differently methylated gene in Table 1), which normally inhibits Wnt signaling, becomes increasingly methylated as OC progresses [46]. As a result, *SOX17* expression is lost, leading to higher β‐catenin levels and its redistribution in the cell. Restoring *SOX17* expression using the demethylating agent 5‐aza2′‐deoxycytidine can suppress TCF/β‐catenin‐dependent transcription and slow down the proliferation of OC cells [46]. On the other hand, when *SOX17* is absent, Wnt signaling remains unchecked, promoting tumor growth [46].

Collectively, these epigenetic alterations demonstrate how aberrant DNAm disrupts multiple components of the Wnt signaling cascade, resulting in sustained proliferative signaling and loss of growth suppressive mechanisms, two of the key Hallmarks of Cancer [44, 45]. Persistent activation of Wnt signaling also promotes tumor cell survival, invasion, and disease progression, emphasizing the biological significance of these DNA methylation events beyond their utility as biomarkers [46].

#### 4.2.2 Cell Cycle and Apoptosis

**FIGURE 4:**
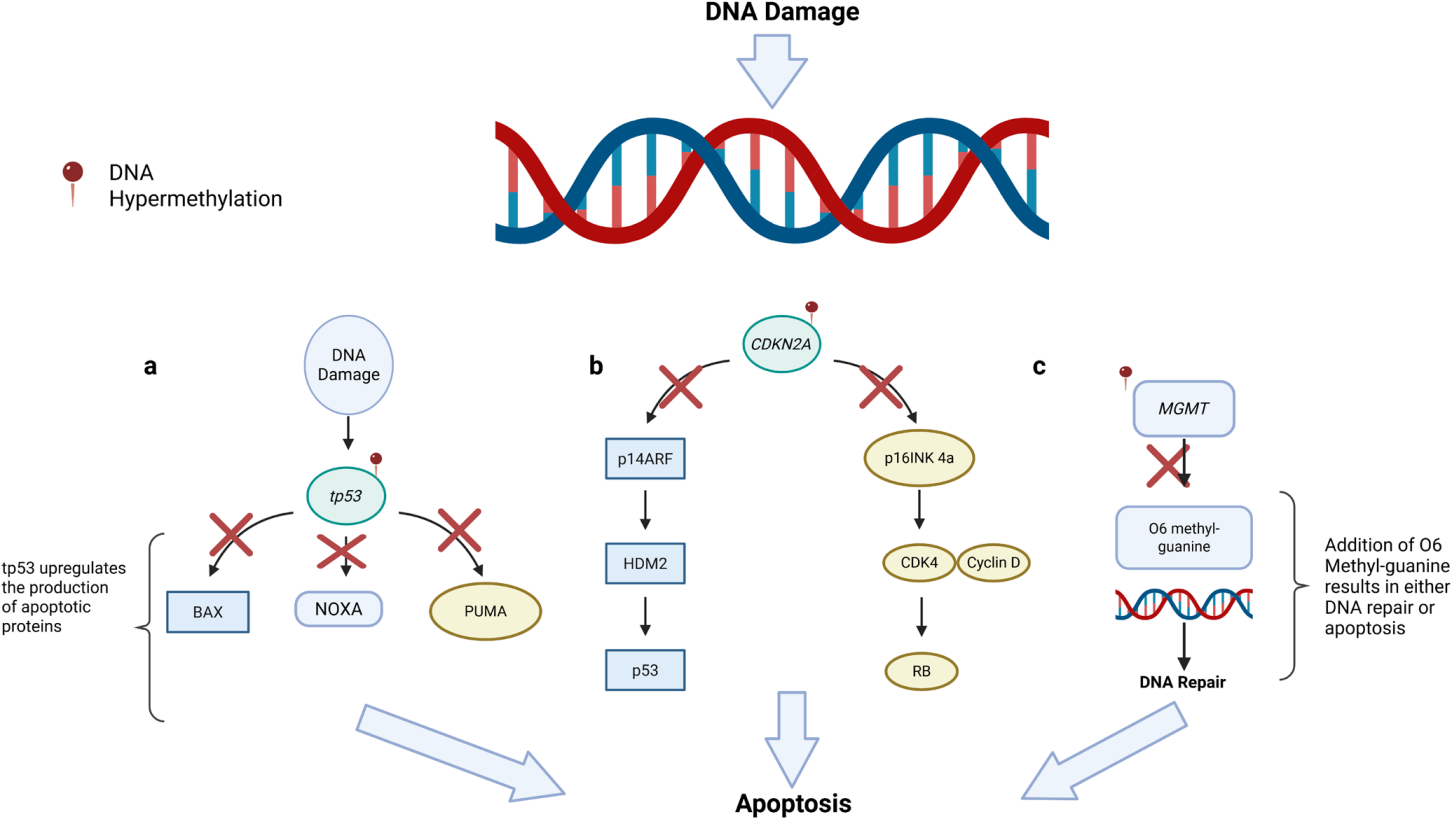
(a) *TP53*, (b) *CDKN2A*, and (c) *MGMT* pathways in their role of promoting apoptosis and their dysregulation in OC. Red lollipop symbols highlight genes that are aberrantly methylated in OC. Created in BioRender, 2025.

*CDKN2A* is one of the first genes to become methylated in the BO to OC transition [30]. *CDKN2A* encodes for two important tumor suppressor proteins, p16INK4a and p14ARF [30]. These proteins regulate the cell cycle and prevent uncontrolled cell proliferation and the loss of function in this gene causes the progression of many cancers [53]. P16INK4a inhibits the activity of cyclin‐dependent kinases 4 and 6, which are needed for the phosphorylation of the retinoblastoma protein (RB1) [54], as illustrated in Figure 4. This process promotes the proliferation of esophageal epithelial cells, contributing to tumor growth. In OC, methylation of *CDKN2A* gene affecting p14ARF results in reduced p53 activity, impairing the cell's ability to undergo apoptosis, which leads to genetically unstable cells contributing to cancer development [53]. When *MGMT* (O‐6‐methylguanine‐DNA methyltransferase) is hypermethylated in cancer, it typically results in silencing of the *MGMT* gene. Under normal circumstances, *MGMT* repairs O‐6‐methylguanine lesions (as seen in Figure 4) in DNA, which are caused by alkylating agents [55]. In esophageal tissue, these DNA lesions may arise through chronic gastroesophageal reflux, inflammation‐associated reactive oxygen and nitrogen species, tobacco smoke and other environmental carcinogens [56]. Promoter hypermethylation of *MGMT* results in transcriptional silencing and impaired DNA repair capacity, allowing alkylated DNA lesions to persist [45]. This repair function is crucial because the accumulation of these lesions can lead to mutations and genome instability, triggering apoptosis or activating other damage response mechanisms [55]. Although loss of *MGMT* expression is well recognized as a predictive biomarker of response to alkylating chemotherapy in glioblastoma [55], in OC its biological significance is more likely related to facilitating tumor initiation and progression through impaired maintenance of genomic integrity rather than treatment response [57]. *TP53* codes for a critical tumor suppressor (p53) that regulates apoptosis through multiple pathways. Upon sensing cellular stress, such as DNA damage, p53 is activated and triggers the transcription of pro‐apoptotic genes, leading to mitochondrial dysfunction and production of pro‐apoptotic proteins such as PUMA and BAX [58], as illustrated in Figure 4. By promoting apoptosis, p53 helps eliminate damaged or potentially cancerous cells, maintaining genomic integrity [59]. Loss or mutation of p53 is a common feature of many cancers, contributing to resistance to apoptosis, genetic instability, and tumor progression in OC [59].

Together, promoter methylation affecting *CDKN2A*, *MGMT*, and *TP53* illustrates how aberrant DNA methylation may disrupt pathways governing cell‐cycle control, DNA repair, and apoptosis. These alterations directly contribute to several Hallmarks of Cancer, including evasion of growth suppressors, resistance to cell death, and genomic instability, thereby facilitating malignant transformation and progression [44, 45]. This further supports the importance of these DNAm markers as both mechanistic drivers of esophageal carcinogenesis and promising biomarkers for disease detection and risk stratification.

## 5 Current Methodological Caveats in OC Epigenetic Research

Epigenetic research in OC has significantly advanced our understanding of the disease. However, there are several methodological caveats of OC epigenetic research that are worth noting. These caveats can impact the interpretation and reproducibility of findings, as well as the translation of research into clinical applications.

### 5.1 Phenotype Assessment and Temporal Dynamics

Phenotype assessment refers to the detailed analysis of the observable characteristics or traits of OC, such as OC subtypes, invasiveness, OC cell characteristics, and protein expression markers [60]. Phenotype assessment in OC is crucial for understanding the disease and developing effective treatments [60]. However, several methodological and interpretative caveats can impact the reliability and validity of these assessments such as sample heterogeneity, interpretation challenges, and temporal dynamics [61]. Interpretation challenges may also affect the phenotype assessments in OC [61]. The functional implications of observed phenotypes can be context dependent [62]. For example, the same molecular alteration might have different effects depending on the cellular environment and the presence of other genetic or epigenetic changes [35]. Phenotypic changes observed in OC might be secondary to other underlying processes rather than direct drivers of disease progression [62]. In terms of temporal dynamics, OC evolves over time and with treatment, and phenotypic characteristics can change as the disease progresses. Cross‐sectional studies may not capture these temporal dynamics, leading to incomplete or misleading conclusions [62]. In order to address some of these issues, studies such as longitudinal cohort studies (to identify DNAm changes associated with OC and BO over a long period of time, from BO to late‐stage cancer) and multi‐omics studies incorporating phenotype correlation (combining omics data across different disciplinaries, in particular epigenomic and histological data with clinical phenotypes) are required [63].

### 5.2 Sample Size

Large scale studies on OC and BO patients continue to be a challenge and this, consequently, affects statistical power, reproducibility, and clinical translation [64]. OC DNA methylation studies tend to have between 20 and 800 samples, with rarer studies obtaining over 1000 samples [65]. These datasets are often stratified by OC subtypes and disease stage, further reducing power for subgroup analyses [65]. In comparison, methylation studies in more prevalent cancers, such as breast [66], colorectal [67], and lung [68], commonly exceed 1000–5000 samples, supported by larger cohorts and better‐established resources. In OC, smaller sample sizes are often attributed to high mortality rates, which limit longitudinal data, and to the difficulty of obtaining tumor tissue [65].

High tumor heterogeneity, as discussed above, requires subgroup analysis which reduces the effective sample size. Small sample groups may also fail to capture subgroup‐ specific DNA methylation differences [69]. DNA Methylation changes can occur based on tumor stage, age, genetic background, and environmental factors which complicates generalisability [69].

Epigenome‐Wide Association Studies (EWAS) using larger patient cohorts (to identify and validate DNA methylation patterns that correlate with specific clinical phenotypes) should be carried out to correct these issues [69]. Several EWAS have been conducted on OAC and OSCC, including the study by Maity and colleagues, which analyzed 50 OAC patient samples alongside 12 control samples [70]. Other studies have examined larger OC cohorts [69, 71], yet there remains a need for even larger, well‐powered EWAS with balanced case–control numbers to strengthen the identification of robust epigenetic markers. A larger patient cohort will aid in detecting biologically relevant DNAm changes for OC and between its subtypes, and to understand how DNAm varies between subgroups of patient groups (e.g., smokers and non‐smokers, cancer stage, and sex) [65].

### 5.3 Tissue Heterogeneity

Epigenetic studies are confined by several biological and methodological design issues, in particular surrounding epigenetic variability in cell types and tissues [72]. Variability between tumors from different patients can also affect phenotype assessment [73]. This variability can be due to genetic differences, environmental factors, and diverse etiological factors, making it challenging to generalize findings [64]. While there is overlap in the DNAm patterns in OSCC and OAC, there are differences in their epigenetic landscapes which may require further subtype DNAm analysis [27]. This variation may lead to the full genetic and epigenetic picture not being captured in studies [27]. In addition to interpatient variability, intratumour heterogeneity represents a further challenge for epigenetic biomarker development in OC. DNA methylation profiling has demonstrated substantial intratumour epigenetic heterogeneity in OC, indicating that different regions of the same tumor may harbor distinct DNA methylation patterns [74]. Increased intratumour DNA methylation heterogeneity has been associated with lymph node metastasis and poorer overall survival, suggesting that epigenetic diversity contributes to tumor progression and may influence biomarker performance [74]. Consequently, reliance on a single tumor biopsy may fail to capture the complete epigenetic landscape of an individual tumor, highlighting the importance of approaches such as multi‐region sampling [64], single‐cell methylation profiling [75], and liquid biopsy to better characterize tumor‐wide epigenetic alterations. Although most evidence for intratumour DNA methylation heterogeneity currently comes from OSCC, similar challenges are likely to be relevant to OAC given its well‐recognized genomic and clonal heterogeneity.

To overcome this caveat, several algorithms and techniques can be used including Surrogate Variable Analysis (SVA), single‐cell DNA methylation profiling, and tissue microdissection [76]. SVA is a statistical method used to identify hidden or unobserved variables that might be driving variations in high‐dimensional data, such as OC or BO DNA methylation, that are not due to the experimental conditions or biological factors of interest [76]. These variables can then be used to adjust the analysis, removing their effects and enabling more accurate downstream analyses [76]. By doing so, it helps researchers identify biologically relevant signals in OC and BO tissue samples by correcting for confounding factors that are not directly measured [77]. Single‐cell DNA methylation profiling enables the examination of DNA methylation at the single‐cell level, which is important for understanding cellular heterogeneity in OC tissues where different cell types may have distinct methylation profiles, potentially influencing OC and BO mechanisms [75]. Like phenotype assessment issues, these caveats could also be addressed using longitudinal studies to assess DNAm changes over time, multi‐omic studies and multi‐region biopsy studies to get a more comprehensive view of the epigenetic patterns [64].

### 5.4 DNA Methylation Profiling

DNA methylation profiling is a powerful tool for understanding epigenetic changes in OC, however there are issues to be kept in mind such as technical variability, data analysis and interpretation, and applications in clinical settings [78]. In clinical settings, identifying robust and clinically relevant DNA methylation biomarkers requires extensive validation across diverse patient cohorts [78]. Variability in patient populations, sample collection, and processing methods can impact biomarker performance. There are several issues to consider while comparing various methods such as the different sensitivities, specificities, and coverage of various methods for DNA methylation profiling (e.g., bisulfite sequencing, DNA methylation arrays, and methylation‐specific PCR) [79]. Results can vary depending on the chosen method. Technical variability between different batches of samples or reagents can introduce bias [79]. While most OC methylation studies are carried out with technologies such as the 450 K or the EPIC array, the new EPIC 2.0 array, whole genome bisulfite sequencing (WGBS), and single‐cell analysis are more advanced technologies that can significantly enhance our understanding of the role of epigenomics in OC [80]. The EPIC 2.0 array offers a more comprehensive and refined set of probes for capturing DNA methylation changes in OC, improving on previous arrays like the 450 k [80]. This enhanced coverage allows for more accurate detection of DNA methylation alterations across the genome, including regions previously underrepresented in earlier arrays [80]. WGBS offers base‐pair resolution of DNA methylation patterns in OC and BO, allowing researchers to capture global methylation changes associated with OC progression [81]. Furthermore, single‐cell WGBS helps dissect epigenetic heterogeneity, identifying distinct subpopulations within tumors and the OC subtypes [81]. However, WGBS is expensive to run and requires high DNA input, factors that can make it inaccessible for large‐scale studies or labs with limited resources [82]. More recently, Enzymatic Methyl‐sequencing (EM‐seq) has emerged as an alternative to conventional bisulfite sequencing [83]. Unlike bisulfite conversion, EM‐seq employs enzymatic reactions to distinguish methylated from unmethylated cytosines while preserving DNA integrity [83]. This results in improved sequence quality, reduced DNA degradation and increased library complexity, making it particularly advantageous for low‐input clinical samples such as circulating cell‐free DNA and FFPE‐derived DNA [83]. As the technology becomes an emerging alternative to conventional bisulfite sequencing, EM‐seq represents a promising approach for high‐resolution methylation profiling in OC.

Most OC studies rely on bisulfite sequencing or DNA methylation arrays, which may misinterpret hydroxymethylation as true methylation and lack single‐cell resolution [84]. While differentially methylated regions (DMRs) are widely reported, their functional relevance is often unclear due to the limitations of bulk sequencing [84], tumor heterogeneity, and Formalin‐Fixed Paraffin‐Embedded (FFPE) related artifacts [85]. Future studies should integrate single‐cell DNA methylation sequencing, hydroxymethylation‐specific methods, and functional assays to confirm true epigenetic changes in OC.

## 6 Future Perspectives

Most cancer DNA methylation studies have focussed on candidate gene studies or array‐based methods using a standard EWAS design. However, researchers are increasingly using machine learning (ML) applications in epigenomic‐based biomarkers. ML automates the process of extracting patterns from data with supervised ML learning a model that captures the relationship between input data and the outcome variable [86]. There has been significant interest in applying predictive ML‐based solutions, particularly supervised ML, for the detection and classification of OC and BO from DNA methylation (DNAm) data [24, 71, 87]. DNAm data is often high dimensional with models suffering from the curse of dimensionality, high computational complexity, and overfitting [88]. As such, ML techniques are commonly employed to mitigate high dimensionality via feature reduction. Differential methylation is the most abundantly used approach [31, 42, 89], but the range of employed ML feature reduction methods is expanding, for example, Boruta [90], and backward‐elimination [91]. Furthermore, unsupervised ML, particularly clustering methods such as PCA [92], or kmeans [93], have been applied to discover OC molecular subtypes which can aid in prediction of response to therapies and overall survival. Meta‐ analysis is a statistical method which can combine data across multiple studies and cohorts [94]. This allows identification of heterogeneity for a more accurate quantification and combining study data will aid in overcoming the limitation of small sample size [94]. This method has also proved useful in identifying results to be prioritized for further analysis and studies [94].

Circulating tumor DNA (ctDNA) is single or double stranded DNA that has been released into blood by apoptotic tumor cells [95]. ctDNA can be used in the form of a non‐invasive liquid biopsy for early detection, treatment response assessment, and residual disease detection [95]. Recently, studies have investigated the diagnostic value of circulating cell‐free DNA (cfDNA) and circulating tumor DNA (ctDNA) obtained through liquid biopsy for the detection of early‐stage OC [96]. This method would be optimal over the current OC diagnostics, as it is non‐invasive and gives insights into tumor epigenetics without the need for procedures such as endoscopy and biopsy [9]. Liquid biopsy in OC may serve to aid in the early detection of OC and assess risk of OC in patients with BO through ctDNA methylation patterns [9]. Several circulating methylated DNA biomarkers have demonstrated promising diagnostic potential, with aberrant methylation of genes including *SHOX2*, *SEPTIN9*, *RNF180*, and *EPO* detected in plasma‐derived ctDNA and shown to distinguish patients with esophageal cancer from healthy controls [96]. Furthermore, multi‐gene methylation panels have demonstrated improved diagnostic performance compared with individual biomarkers, suggesting that combining multiple DNA methylation targets may enhance the sensitivity and specificity of minimally invasive early detection strategies [96]. However, despite these encouraging findings, further prospective validation studies and assay standardization are required before blood‐based methylation biomarkers can be routinely implemented in clinical practice. A potential cfDNA marker in a liquid biopsy is examination of 5hmC as the patterns, specifically the loss, of 5hmC has been shown to indicate carcinogenesis and could potentially correlate with cancer stage and treatment options in OC [97].

In addition to blood‐based biomarkers, minimally invasive esophageal sampling devices coupled with DNA methylation assays have shown considerable promise for BO detection [98]. The EsoCheck device enables non‐endoscopic collection of esophageal epithelial cells, while the companion EsoGuard assay analyses methylation of genes including *VIM* and *CCNA1* to identify BO with high sensitivity and specificity (> 90%) [98]. These technologies offer an alternative to conventional endoscopy for screening at‐risk populations and may improve early detection of BO and OAC [99]. Nevertheless, further prospective validation and cost‐effectiveness studies are required before widespread clinical implementation.

Multi‐omic profiling is the integration of data from genomics, epigenomics, proteomics, transcriptomics and other omic areas and this is useful in complex diseases, such as OC, that are driven by alterations in multiple biological processes [82]. This creates a need for a comprehensive and integrative approach for the development of biomarkers for diagnostic, therapeutic, or prognostic purposes. Biomarkers from different omics data can lead to improved diagnostics with better specificity and sensitivity and may allow novel biomarkers to be discovered through the integration of data [63]. For OC, there are several studies taking a multi‐omics approach to discovering novel therapeutic and diagnostic markers such as Zhao and colleagues, combining data from omics such as epigenomics, proteomics, transcriptomics and phosphoproteomics [100]. Multi‐omic profiling in OC and BO can also contribute to capturing the dynamics of tumor evolution, treatment response and resistance mechanisms over time which will contribute to our overall knowledge of BO and OC [100]. Beyond DNAm alone, studies have explored the benefits of multimodal approaches, leveraging ML to extract richer and more diverse information in a multi‐omics setting (e.g., mRNA), gene expression and DNA fragmentation markers [81], potentially leading to more efficacious prediction models. Due to the large variability of DNAm in OC, combining a panel with another method of diagnostics/prognostics, such as specific aptamers, mutation screening, imaging and biopsy, could potentially be beneficial in increasing the specificity and sensitivity of a potential diagnostic assay.

## 7 Conclusion

DNA methylation has emerged as one of the most promising epigenetic mechanisms for the identification of biomarkers capable of improving the early detection, diagnosis, and risk stratification of OC. Numerous methylation alterations have been identified in BO, OAC, and OSCC, with several candidate biomarkers and multi‐marker panels demonstrating encouraging diagnostic and prognostic performance. However, considerable heterogeneity in study design, patient cohorts, analytical platforms, and validation strategies has limited direct comparison between studies and delayed clinical translation. Advances in genome‐wide methylation profiling, minimally invasive sampling approaches, machine learning, and multi‐omics integration provide new opportunities to improve biomarker discovery and develop more robust, clinically applicable methylation panels. Future studies should prioritize large, well‐characterized prospective cohorts and standardized analytical methodologies to enable independent validation and facilitate implementation into clinical practice. Collectively, DNA methylation biomarkers remain a highly promising approach for improving the prevention, early detection, and personalized management of OC.

## Author Contributions

L.L. performed the literature search, interpretation of the results and drafted the manuscript. T.D. performed a literature search for machine learning (ML) applications in epigenomic‐based biomarkers and drafted the manuscript. C.S. and N.G. drafted and reviewed the manuscript. T.M.M. drafted and reviewed the manuscript and was involved in the overall manuscript conceptualization. All authors reviewed and approved the final manuscript.

## Funding

This work was supported by the Fiosraigh Scholarship Programme by the Dean of Graduate Research School, TU Dublin.

## Ethics Statement

The authors have nothing to report.

## Conflicts of Interest

T.M.M. and T.D. are co‐inventors on a pending patent pertaining to an epigenetic‐based diagnostics test for Esophageal Cancer. All other authors have declared that no competing interests exist.

## Data Availability

Data sharing not applicable to this article as no datasets were generated or analyzed during the current study.
